# Design, Manufacturing, Tribology, and Performance of Microgears and Microgear Trains: A Critical Review of Mechanical Power Transmission at the Microscale

**DOI:** 10.3390/mi17080934

**Published:** 2026-08-05

**Authors:** Ioan Doroftei, Cristina-Magda Cazacu

**Affiliations:** 1 Mechanical Engineering, Mechatronics and Robotics Department, “Gheorghe Asachi” Technical University of Iasi, 43 D. Mangeron Blvd, 700050 Iasi, Romania; 2 Technical Sciences Academy of Romania, 26 Dacia Blvd, 030167 Bucharest, Romania; 3 Academy of Romanian Scientists, 3 Ilfov, 050044 Bucharest, Romania

**Keywords:** microgear, microgear train, mechanical power transmission, MEMS, microfabrication, microtribology, transmission efficiency, microsystems

## Abstract

Microgears enable mechanical power transmission, speed reduction, motion conversion, and synchronization in compact devices ranging from microelectromechanical systems to miniature robots and optically driven micromachines. Their behavior cannot, however, be inferred by geometrically scaling conventional gears alone. As size decreases, relative manufacturing errors, surface forces, friction, adhesion, environmental sensitivity, and metrological uncertainty become increasingly important, while torque capacity and stored kinetic energy decrease rapidly. This critical review integrates the design, manufacture, tribology, and system-level performance of microgears and microgear trains. It first clarifies dimensional terminology and derives the principal scaling relationships. It then compares external, internal, planetary, worm, bevel, compliant, and reconfigurable transmission architectures; evaluates silicon micromachining, electroforming, micro powder injection molding, microforming, micro-electrical discharge machining, ultrashort-pulse laser ablation, and additive microfabrication; and examines adhesion, friction, wear, lubrication, and environmental effects. Particular attention is paid to transmission efficiency, starting torque, backlash, transmission error, lifetime, and the influence of the measuring instrument on the observed response. The literature remains strongly weighted toward manufacturability and isolated components, whereas reproducible, loaded, system-level tests are comparatively scarce. On this basis, the review proposes a unified hierarchy of validation, a minimum functional test matrix, and scale-aware design indicators. The central conclusion is that successful microgear transmissions require concurrent design of geometry, process, surface condition, environment, load path, and measurement strategy.

## 1. Introduction

Miniaturization has transformed sensing, actuation, fluid handling, medical instrumentation, and robotics by integrating mechanical functions with electronics and microfabricated structures [1]. Within this landscape, gears remain attractive because they provide a deterministic geometric relationship between input and output motion, can multiply torque without continuous feedback, and can distribute motion among several elements. Microgears have, consequently, appeared in microelectromechanical systems (MEMSs), miniature pumps, microrobots, optical manipulation platforms, and experimental metamachines [2,3,4].

The term microgear covers markedly different objects. Some are planar silicon gears with tooth dimensions of a few micrometers; others are metallic or ceramic gears with modules near 0.1 mm and overall diameters of several millimeters. They may transmit appreciable mechanical power, operate intermittently as indexing elements, or merely constrain the relative motion of optically or magnetically driven particles. A useful review must therefore distinguish geometric scale from functional role and fabrication route. VDI 2731 provides an umbrella vocabulary for microgears [5], whereas the flank-tolerance framework of ISO 1328-1 remains relevant to individual tooth geometry but does not by itself characterize a complete microscale transmission [6].

Three features make microscale gearing a distinct engineering problem. First, geometric similarity does not preserve the balance between available torque, inertia, friction, and adhesion. Second, process-induced deviations and surface roughness occupy a larger fraction of tooth size. Third, torque and displacement are sufficiently small that probes, suspensions, wiring, fluid drag, and optical tracking can modify the behavior being measured. Silicon wear studies, surface-functionalization experiments, and starting-torque measurements demonstrate that contact conditions and instrumentation can be as decisive as nominal involute geometry [7,8,9].

Existing reviews have mapped the manufacture and metrology of miniature and microgears in considerable detail [2]. More recent studies have introduced improved electroformed thickness uniformity [10], parallel two-photon fabrication [11], hybrid tooth-profile measurement [12], and magnetic or optical actuation of functional gear assemblies [3,13]. Nevertheless, design, process capability, microtribology, and measured transmission performance are often discussed separately. This fragmentation obscures the causal chain from a manufacturing decision to contact mechanics, loss, reliability, and application-level function.

The objective of this article is to provide a critical, system-oriented synthesis of mechanical power transmission by microgears. The review (i) establishes working dimensional definitions; (ii) derives scale-aware design indicators; (iii) compares transmission architectures and manufacturing routes; (iv) integrates friction, adhesion, wear, lubrication, and environment; (v) evaluates performance metrics and test methods; and (vi) identifies minimum evidence required to claim a functional microgear transmission. The intended outcome is not a universal miniaturized version of conventional gear design, but a framework for selecting compatible geometry, material, process, surface, environment, and measurement strategies.

## 2. Review Methodology and Scope

### 2.1. Review Design

A critical-review design was adopted because the available evidence spans incompatible length scales, materials, actuation methods, and validation levels. The corpus included journal articles, conference contributions where they reported otherwise unavailable process evidence, and relevant engineering standards. Searches combined terms such as microgear, miniature gear, MEMS gear, microgear train, micro planetary gear, microtribology, starting torque, transmission efficiency, backlash, transmission error, and the names of major fabrication processes. Backward and forward citation tracing were used to connect process-oriented studies to functional transmission studies.

### 2.2. Eligibility and Classification

A publication was retained when it addressed at least one causal element of a physical microscale gear system: geometry, fabrication, material, tooth-contact behavior, measurement, or application-level operation. Studies limited to macroscale gears without an explicit scaling argument were excluded. Purely conceptual mechanisms without fabricated or quantitatively simulated gear contacts were used only when they clarified an architecture. Table 1 summarizes the eligibility criteria.

The extracted evidence was classified along six axes: characteristic size, gear architecture, material, manufacturing route, operating medium, and validation level. This classification permits comparison without implying that a silicon MEMS gear and a millimeter-scale ceramic gear are interchangeable. Numerical results were interpreted in their original test conditions; cross-study rankings were avoided when load, speed, environment, or uncertainty differed materially.

### 2.3. Evidence Synthesis and Limitations

The synthesis follows the transmission chain from input actuation to output function. Particular weight is given to studies that connect at least two levels, for example, process quality and tooth geometry [10,12], surface treatment and wear [8,14], or starting torque and measurement uncertainty [9,15]. The review is not a statistical meta-analysis: datasets are too heterogeneous, and many papers report demonstrators rather than standardized endurance tests. Accordingly, the proposed indicators and test matrix are engineering recommendations derived from convergent evidence, not new standards.

### 2.4. Descriptive Distribution of the Reviewed Evidence

To quantify the thematic imbalance identified during the critical synthesis, a descriptive mapping of the reviewed evidence was performed. This analysis was intended to characterize the composition of the corpus assembled for the present review rather than to provide an exhaustive bibliometric representation of the entire microgear literature. Of the 40 references included in the revised manuscript, 28 were classified as primary technical studies belonging to the microgear evidence corpus analyzed in this review. The general miniaturization study [1], the previous review article [2], the four engineering standards [5,6,16,17], and the general review of stiction in MEMSs and NEMSs [18] were retained as contextual or normative sources but were excluded from the descriptive count. The five additional methodological studies [19,20,21,22,23], introduced to support the discussions of data-driven compensation, digital twins, and ultrashort-pulse laser processing, were also excluded because they address transferable manufacturing and model-updating methodologies rather than microgear-specific experimental evidence.

**Figure 1:**
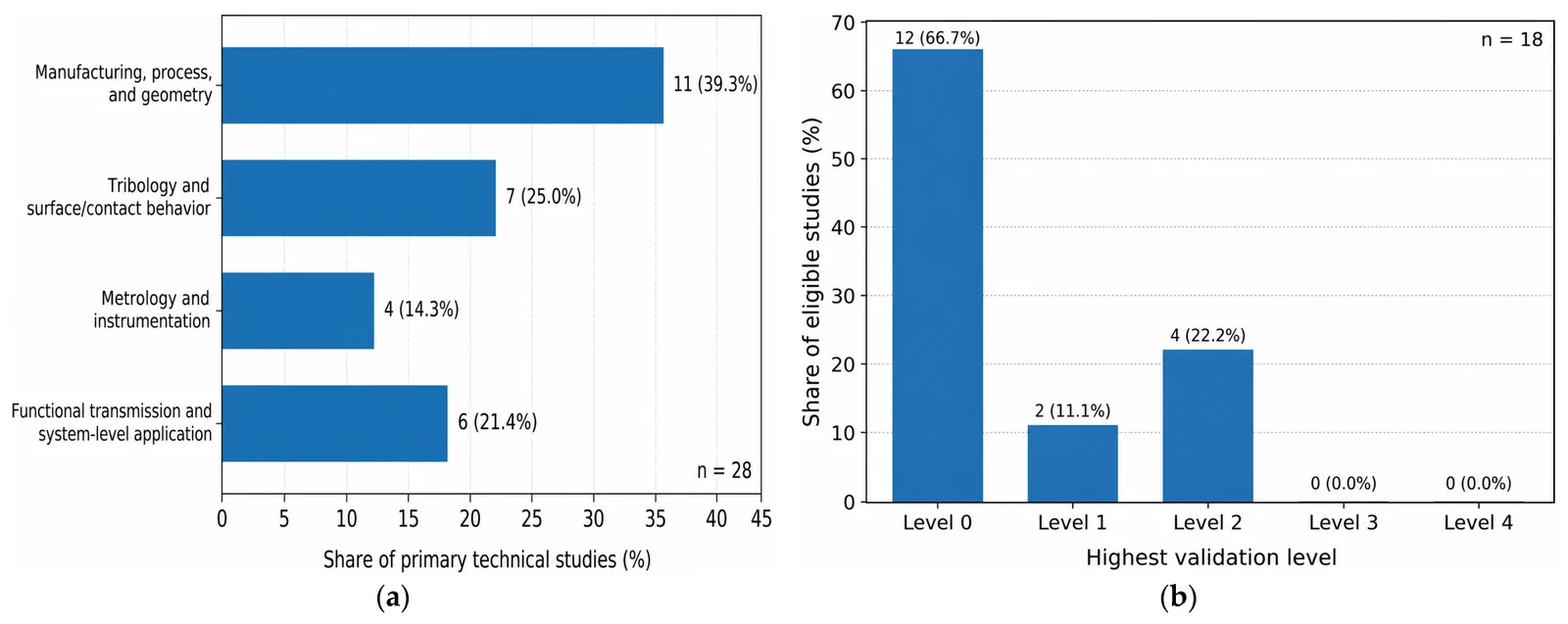
Descriptive distribution of the reviewed technical evidence: (**a**) distribution of the 28 primary technical studies according to their dominant technical emphasis; (**b**) distribution of the 18 eligible studies according to the highest validation level reached. Absolute numbers and corresponding percentages are reported for each category.

Each primary study was assigned to one dominant technical category according to its principal objective and reported evidence. The four categories were: manufacturing, process, and geometry; tribology and surface/contact behavior; metrology and instrumentation; and functional transmission or system-level application. A single dominant category was used for each study to avoid double counting, even when secondary contributions extended into adjacent areas. The detailed classification and the references assigned to each category are reported in Table 2, while the corresponding distribution is visualized in Figure 1a.

Among the 28 primary technical studies, 11 studies (39.3%) were primarily concerned with manufacturing, process development, or geometric characterization; 7 studies (25.0%) focused mainly on tribology and surface/contact behavior; and 4 studies (14.3%) addressed metrology or instrumentation. Only 6 studies (21.4%) were primarily directed toward functional transmission or system-level application. Consequently, 22 of the 28 studies (78.6%) concentrated predominantly on manufacturing, geometry, tribology, or measurement, whereas slightly more than one-fifth of the corpus focused principally on the operation of a complete transmission or microgear-based system.

A second classification was performed using the validation hierarchy introduced in Section 7.4. This analysis was restricted to the 18 studies that reported either a fabricated microgear geometry or the identifiable operation of a microgear-based pair, train, or application system. The remaining 10 primary studies provided important component-level, tribological, simulation, sensing, or calibration evidence, but they were not assigned to Levels 0–4 because they did not directly validate a fabricated microgear geometry or the operation of a microgear transmission.

The highest validation level reached by each of the 18 eligible studies is summarized in Figure 1b. Twelve studies (66.7%) reached Level 0 by demonstrating fabricated geometry and dimensional or process characterization. Two studies (11.1%) reached Level 1 through unloaded rotation or demonstrated meshing. Four studies (22.2%) reached Level 2 by providing quantified functional or loaded-operation evidence. None of the reviewed studies satisfied the complete evidence requirements defined here for Level 3, which combines quantified transmission performance, motion-quality metrics, uncertainty, and repeated testing, or for Level 4, which additionally requires endurance monitoring and failure analysis over a declared operating envelope.

The two distributions support the qualitative assessment developed throughout this review. The available literature provides substantial evidence regarding manufacturability, surface behavior, dimensional quality, and isolated functional demonstrations, but considerably less evidence on reproducible, loaded, uncertainty-qualified, and long-duration transmission performance. This descriptive result does not imply that manufacturing, tribological, or metrological studies are of secondary importance. Rather, it shows that the causal chain linking process, geometry, surface condition, transmitted load, motion quality, efficiency, and degradation is rarely examined within a single experimental framework. The proposed validation hierarchy, minimum test matrix, and reference functional test artifact are intended to help close this gap.

## 3. Definitions, Dimensional Criteria, and Scaling Effects

### 3.1. Working Dimensional Definitions

Gear size may be represented by outside diameter, pitch diameter, face width, tooth height, module, or minimum feature size. Module m is especially useful because it links pitch diameter d and tooth number z through Equation (1):

(1) m = d/z,

Equation (1) is necessary but insufficient: two gears with the same module can differ strongly in diameter, thickness, aspect ratio, and manufacturing route. The working classification in Table 3, therefore, uses both module and overall size. Its boundaries are descriptive rather than normative and should always be accompanied by the actual dimensions.

### 3.2. Kinematic Similarity

For ideal external gears, the angular-speed ratio $i_{12}$ depends on tooth numbers, $z_{1}$ and $z_{2}$, rather than absolute scale, as expressed by Equation (2), where the negative sign denotes opposite rotation. Thus, geometric miniaturization can preserve nominal kinematics:

(2) $$i_{12}\;=\;\omega_{1}/\omega_{2}\;=\;-z_{2}/z_{1},$$

where $\omega_{1}$ and $\omega_{2}$ are the angular speeds of gears 1 and 2.

Real transmissions depart from Equation (2) through backlash, profile error, eccentricity, elastic deflection, intermittent contact, friction, and shaft or bearing compliance. These departures do not necessarily scale with module, so nominal kinematic similarity must not be confused with dynamic or energetic similarity.

### 3.3. Mechanical Scaling

Consider a geometrically similar family in which all lengths scale by a factor λ, as defined by Equation (3):

(3) $$L\;=\;\lambda L_{0}.$$

In Equation (3), L is a generic scaled length, $L_{0}$ is the corresponding reference length, and λ is the geometric scale factor. Under geometric similarity, area scales with $\lambda^{2}$, volume and mass with $\lambda^{3}$, and mass moment of inertia with $\lambda^{5}$.

Following the Lewis-type representation of tooth-root bending and the dependence of bending capacity on tangential load, face width, module, and tooth-form factors used in standardized gear-strength formulations [16,17], a simplified first-order tooth-root capacity can be written as Equation (4):

(4) $$F_{t,allow}\;≃\;\sigma_{allow}\;b\;m\;Y;\;\;\;F_{t,allow}(\lambda )/F_{t,allow}(1)\;≃\;\lambda^{2}.$$

In Equation (4), $F_{t,allow}$ is the allowable tangential tooth force, $\sigma_{allow}$ is the allowable stress, b is the face width, m is the module, Y is a tooth-form factor, and λ is the scale factor. The notation $F_{t,allow}$(λ) denotes the allowable force at scale λ, while $F_{t,allow}$(1) denotes the reference value at λ = 1. Under similarity, b and m each scale with λ, so the allowable tangential force scales approximately with $\lambda^{2}$.

Equation (4) is used here only as a first-order scaling relation and is not intended to replace a complete ISO or AGMA tooth-bending-strength calculation. Dynamic effects, load distribution, stress concentration, rim thickness, size effects, manufacturing deviations, and reliability factors are deliberately omitted so that the geometric scaling exponent can be isolated. Under the assumed geometrical similarity, the material allowable stress and the tooth-form factor are held constant, whereas both b and m vary linearly with λ.

Because the pitch radius also scales with λ, the corresponding torque capacity follows Equation (5):

(5) $$T_{allow}\;=\;F_{t,allow}\;d/2;\;\;\;T_{allow}(\lambda )/T_{allow}(1)\;≃\;\lambda^{3}\;.$$

In Equation (5), $T_{allow}$ is the allowable torque, $F_{t,allow}$ is the allowable tangential tooth force, and d/2 is the pitch radius. The terms $T_{allow}$(λ) and $T_{allow}$(1) denote the torque capacity at the scaled and reference sizes, respectively. The available torque therefore decreases approximately with the cube of scale.

Rotational inertia decreases even faster, as stated in Equation (6):

(6) $$J(\lambda )/J(1)\;≃\;\lambda^{5}\;.$$

In Equation (6), J(λ) is the mass moment of inertia at scale λ, and J(1) is the reference inertia. The $\lambda^{5}$ dependence is beneficial for rapid acceleration, but it also means that small disturbance torques, adhesion, or probe forces can dominate transient response. The principal scaling relations are consolidated in Table 4. They are first-order guides; thin-film structures, non-similar face widths, size-dependent material behavior, and fabrication constraints can modify the exponents.

### 3.4. Relative Errors and Surface-Dominated Effects

A dimensional deviation becomes functionally meaningful relative to tooth size. Equation (7) defines representative normalized indicators for profile deviation, backlash, and arithmetic roughness:

(7) $$\varepsilon_{\alpha }\;=\;f_{\alpha }/m,\;\;\;\beta \;=\;j/m,\;\;\;\rho_{a}\;=\;R_{a}/m\;.$$

In Equation (7), $\varepsilon_{\alpha }$ is the normalized profile-deviation indicator, $f_{\alpha }$ is the absolute profile deviation, β is the normalized backlash indicator, j is backlash, $\rho_{a}$ is the normalized roughness indicator, $R_{a}$ is the arithmetic mean roughness, and m is the module. A process that holds an absolute error nearly constant will exhibit increasing normalized error as module decreases. Equation (7) explains why apparently small burrs, edge radii, taper, and roughness can strongly alter contact ratio and load sharing. Hybrid profile metrology has been proposed precisely because no single optical or tactile method reliably captures all tooth regions at these scales [12]. Thickness nonuniformity is similarly consequential in electroformed gears because it changes face contact, stiffness, and out-of-plane alignment [10].

Surface forces scale differently from bulk forces. Capillary, electrostatic, van der Waals, and chemically mediated adhesion may therefore control starting behavior even when they contribute little during steady motion. Stiction in MEMSs is a system-level failure mode rather than merely a high coefficient of friction [18]. Hydrophobic and low-friction films can reduce adhesion, but their effectiveness depends on load, humidity, contamination, and durability [33].

### 3.5. Operating Medium

The surrounding medium is part of the transmission. In air, humidity can create capillary bridges and accelerate tribochemical reactions; in vacuum, adsorption and heat removal change; in liquids, viscous drag may suppress impacts while adding a speed-dependent loss. The Reynolds number in Equation (8) indicates whether inertial or viscous effects dominate the external flow:

(8) $$Re\;=\;\rho_{f}\;U\;L/\mu_{f}.$$

In Equation (8), Re is the Reynolds number, $\rho_{f}$ is the fluid density, U is a characteristic velocity, L is a characteristic length, and $\mu_{f}$ is the dynamic viscosity. At small L, Equation (8) commonly yields low Reynolds numbers, making viscous forces repeatable but potentially large relative to available torque. The implication of Section 3.2, Section 3.3, Section 3.4 and Section 3.5 is direct: a microgear should be specified by its dimensions, architecture, material, process, surface condition, medium, load, and speed, not by diameter or module alone.

## 4. Microgear Geometries and Transmission Architectures

### 4.1. External and Internal Cylindrical Gears

Planar external spur gears are the dominant microgear architecture because lithography, electroforming, planar molding, and two-photon fabrication naturally generate constant-thickness profiles [10,11,25]. Their ideal angular-speed ratio is given by Equation (2), where $i_{12}$ is the external-mesh transmission ratio, $\omega_{1}$ and $\omega_{2}$ are the angular velocities of the two gears, and $z_{1}$ and $z_{2}$ are their tooth numbers; the negative sign indicates opposite rotation. The architecture is simple to inspect, but external meshing reverses rotation and requires center-distance control.

Internal gearing preserves the direction of rotation and can increase compactness. Its ideal ratio is expressed by Equation (9):

(9) $$i_{12,int}\;=\;\omega_{1}/\omega_{2}\;=\;+z_{2}/z_{1}.$$

In Equation (9), $i_{12,\mathrm{int}}$ is the internal-mesh transmission ratio, while $\omega_{1}$, $\omega_{2}$, $z_{1}$, and $z_{2}$ retain the meanings defined for Equation (2); the positive sign indicates that the two gears rotate in the same direction. At small tooth-number differences, however, interference, undercut, tip clearance, and process-induced edge rounding become restrictive.

### 4.2. Planetary and Compound Gear Trains

Planetary trains combine coaxial input and output, compactness, and potentially high reduction. Willis’ relation is given by Equation (10):

(10) $$(\omega_{s}\;-\;\omega_{c})/(\omega_{r}\;-\;\omega_{c})\;=\;-z_{r}/z_{s}.$$

In Equation (10), $\omega_{s}$, $\omega_{r}$, and $\omega_{c}$ are the angular velocities of the sun gear, ring gear, and carrier, respectively, while $z_{s}$ and $z_{r}$ are the tooth numbers of the sun and ring gears. With the ring fixed, Equation (10) reduces to Equation (11):

(11) $$i\;=\;\omega_{s}/\omega_{c}\;=\;1\;+\;z_{r}/z_{s}.$$

In Equation (11), i is the speed ratio between sun and carrier for the fixed-ring case; $\omega_{s}$, $\omega_{c}$, $z_{r}$, and $z_{s}$ have the meanings defined above. The kinematic advantage is clear, but successful microscale implementation requires simultaneous planet engagement, accurate pin positioning, controlled clearances, and acceptable carrier/bearing friction. At the microscale, shaft and planet-pin positioning should be treated as part of the functional tooth-contact geometry rather than solely as an assembly requirement. Static errors in the coordinates of the shaft or pin centers modify the operating center distance and circumferential phase, while angular misalignment and axial end play reduce the effective face overlap and may promote localized edge contact. Radial bearing clearance permits load-dependent migration of the shaft center within the support, so the operating mesh geometry may differ between unloaded rotation, start-up, and transmitted-load conditions. These effects become particularly significant when the absolute positioning error or clearance represents a non-negligible fraction of the module m or face width b. Accordingly, microgear-train studies should report the nominal and measured shaft-center coordinates, pin-position errors, radial and axial bearing clearances, support stiffness or preload, shaft and gear runout, angular misalignment, and the assembly procedure. Scale-aware reporting should relate shaft- or pin-positioning error and radial bearing clearance to the module, and axial displacement or loss of face overlap to the face width.

In planetary trains, differences among individual planet-pin positions and clearances can produce unequal mesh clearances, imperfect planet phasing, and nonuniform load sharing. A high theoretical ratio does not guarantee useful output torque if several lightly loaded contacts and supports consume the available input. Compound trains can achieve larger ratios with planar stages, but cumulative eccentricity and backlash increase with stage count. In compound trains, shaft-positioning errors, bearing clearances, runout, and backlash accumulate across successive stages and can interact with support compliance, so the measured output response cannot be attributed to tooth geometry alone. Arai’s analysis of microgear-train power transmission shows why each stage must be treated as an energetic element rather than an ideal ratio block [4].

### 4.3. Helical, Bevel, Worm, and Nonconventional Architectures

Helical teeth can improve contact continuity, yet they introduce axial force and require three-dimensional tooth manufacture. Bevel and line gears redirect motion between intersecting or skew axes; a two-step fabrication method for miniature bevel line gears illustrates the process planning required to preserve flank geometry [24]. Such gears are attractive when packaging dominates, but alignment and metrology are more difficult than for planar spur gears.

Worm gearing offers a large reduction in one stage. Its ideal ratio is given by Equation (12):

(12) $$i\;=\;z_{gear}/z_{w}.$$

In Equation (12), i is the worm-gear speed ratio, $z_{gear}$ is the tooth number of the gear, and $z_{w}$ is the number of worm starts. Sliding is inherent, so friction, heat, and wear can erase the compactness advantage. Self-locking should never be inferred from geometry alone at the microscale because friction is environmentally and speed dependent. Compliant or strain-wave concepts may also provide high reduction. Their simplified ratio, when the circular spline is fixed and the wave generator is input, is shown in Equation (13):

(13) $$i\;=\;\omega_{in}/\omega_{out}\;=\;-z_{f}/(z_{c}\;-\;z_{f}).$$

In Equation (13), i is the strain-wave speed ratio, $\omega_{in}$ and $\omega_{out}$ are input and output angular velocities, $z_{f}$ is the flexspline tooth number, and $z_{c}$ is the circular-spline tooth number. Realization at small scale is constrained by fatigue, minimum wall thickness, and three-dimensional process capability. At the opposite end of the design space, optically or magnetically assembled gear particles prioritize reconfigurability rather than high power density. Magnetic femtosecond-written gears [3], optoelectronic-tweezer micromachines [13], and geared colloidal metamachines [38] demonstrate that meshing can coordinate distributed microscale motion even when the gears are not mounted on conventional shafts.

### 4.4. Architecture Selection

Table 5 compares the principal architectures. Selection should begin with output function and packaging, and then test whether the required ratio, torque margin, alignment, fabrication route, and environment can coexist.

## 5. Materials and Manufacturing Technologies

### 5.1. Material–Process–Geometry Coupling

At the microscale, material selection cannot be separated from manufacturing. A process determines not only feature size but also thickness, taper, edge radius, residual stress, texture, surface chemistry, and feasible assembly sequence. These attributes control tooth stiffness and contact behavior. A nominally stronger material can therefore produce a poorer gear if the selected route generates larger normalized deviations or an unsuitable surface.

### 5.2. Silicon Micromachining and Lithographic Routes

Silicon offers mature lithography, batch processing, and integration with MEMS. Planar tooth profiles can be defined accurately, but brittle fracture, limited out-of-plane thickness, and tribological vulnerability restrict loaded dry contact. Wear experiments on silicon MEMS surfaces show strong dependence on contact pressure, environment, and surface condition [7]. Conformal self-assembled layers can reduce adhesion, yet local coating damage on silicon microgears may create abrupt changes in friction and wear [8].

Lithography followed by electroforming enables thicker metallic gears and avoids the brittleness of monolithic silicon. The process can yield fine in-plane geometry, but current density, mass transport, seed layers, resist sidewalls, and release govern thickness uniformity and residual stress. Multi-step self-aligned lithography and electroforming improved microgear thickness uniformity, illustrating that out-of-plane quality must be designed rather than treated as a secondary tolerance [10]. Projection lithography similarly supports efficient replication of planar gear patterns [25].

### 5.3. Molding, Forming, and Replication

Micro powder injection molding (micro-PIM) is attractive for batch manufacture of metallic and ceramic gears. It combines mold-defined shape with debinding and sintering, but shrinkage, warpage, feedstock homogeneity, and demolding can alter tooth geometry. Characterization of molded microgears has shown that replication quality depends on both filling and downstream thermal processing [26]. Zirconia microgear studies further demonstrate that feedstock and process parameters govern moldability and final defects [27]. Recent zirconia–alumina work links fabrication conditions to tooth-profile integrity and mechanical characterization [28].

Microforming can provide favorable material utilization and production rate. A process chain for module 0.1 mm gears combines forming operations while explicitly managing tooling and geometry [29]. Piezoelectrically actuated microblanking offers a compact means of delivering controlled strokes and forces [30]. Both routes face size effects in friction, material flow, grain structure, die alignment, and burr formation; the resulting edge condition may be as important as conventional pitch or profile error.

### 5.4. Subtractive and Additive Microfabrication

Micro-cutting and micro-electrical discharge machining (micro-EDM) provide material flexibility and three-dimensional access. Tool runout, minimum edge radius, tool wear, recast layers, and debris removal are limiting factors. Micro-wire EDM has produced gears with intact tooth profiles when discharge and path parameters were coordinated [31]. Subtractive processes are well suited to functional metallic prototypes but may be less economical for large batches than replication routes.

Ultrashort-pulse laser ablation provides a complementary non-contact subtractive route for metallic and ceramic microgears. Picosecond and femtosecond systems can machine microholes and three-dimensional microfeatures in metals, alloys, and ceramics without mechanical tool wear, although the attainable removal rate and surface integrity depend strongly on pulse duration, fluence, repetition rate, spot overlap, scanning strategy, focusing conditions, and material response [23]. A gear-specific demonstration was reported by Uppal et al., who used femtosecond laser micromachining to fabricate a three-dimensional hard-PZT microgear with an approximate diameter of 1.2 mm and a tooth size of approximately 0.2 mm; the piezoelectric properties were reported to remain unaffected by the optimized process [32]. For microgear manufacture, this route is particularly attractive for hard, brittle, or otherwise difficult-to-machine materials and for the rapid prototyping of complex contours. Its limitations include parameter-sensitive edge quality and taper, redeposition or debris, residual roughness, possible microcracking or localized thermal alteration, and lower throughput than replication-based processes. Accordingly, the laser-processed geometry should be evaluated through the normalized profile, roughness, and backlash indicators introduced in Equation (7), namely $\frac{f_{\alpha }}{m}$, $\frac{R_{a}}{m}$, and $\frac{j}{m}$, rather than through successful shape generation alone.

Two-photon polymerization and related direct-write techniques allow complex three-dimensional gears, integrated shafts, and unconventional topologies. Parallel photopolymerization increases throughput [11], while femtosecond writing can produce magnetically actuated gear pairs [3]. Additive freedom is offset by voxel anisotropy, polymer creep, shrinkage, roughness, and limited torque capacity. These processes are especially valuable for low-load devices and architectures that cannot be released or assembled by planar methods.

### 5.5. Comparative Assessment and Design for Manufacture

Table 6 compares the main material–process combinations. The most appropriate process is the one whose characteristic deviations remain acceptable relative to module, face width, and clearance, while also providing the required surface state and production volume.

A scale-aware design-for-manufacture sequence is therefore recommended: select the process window; establish minimum tooth, gap, thickness, and release features; allocate profile, pitch, runout, taper, and roughness budgets; simulate the as-manufactured rather than nominal geometry; and plan inspection before finalizing inaccessible internal or stacked features. This sequence reverses the common practice of selecting an ideal gear first and searching afterward for a process capable of reproducing it.

## 6. Microtribology: Friction, Adhesion, Wear, and Lubrication

### 6.1. From Coulomb Friction to Adhesive Contact

The conventional Coulomb relation $F_{f}$ = μN is incomplete when adhesion contributes an effective normal load. A useful engineering representation is Equation (14):

(14) $$F_{f}\;=\;\mu (N\;+\;F_{adh}).$$

In Equation (14), $F_{f}$ is the friction force, μ is the coefficient of friction, N is the externally applied normal load, and $F_{adh}$ is the adhesive contribution to the normal load, including capillary, van der Waals, electrostatic, and chemically mediated effects. Equation (14) explains why a nominally unloaded microgear may require a finite starting torque and why friction does not necessarily scale with transmitted tooth force. Adhesion and friction measurements on silicon and low-friction films confirm strong scale and surface-state effects [33]. In stationary contact, humidity-dependent capillary bridges and contamination can cause stiction; during motion, plowing, interfacial shear, and third-body particles contribute to loss.

### 6.2. Loss Mechanisms

A system-level power balance should include tooth sliding/rolling, supports, fluid drag, seal or confinement forces, and actuation coupling. Equation (15) expresses this decomposition:

(15) $$P_{loss}\;=\;P_{mesh}\;+\;P_{support}\;+\;P_{fluid}\;+\;P_{aux}.$$

In Equation (15), $P_{loss}$ is the total power loss, $P_{mesh}$ is the mesh loss, $P_{support}$ is the support or bearing loss, $P_{fluid}$ is the fluid-drag loss, and $P_{aux}$ represents auxiliary losses associated with seals, confinement, actuation coupling, or measurement interfaces. Transmission efficiency then follows Equation (16):

(16) $$\eta \;=\;P_{out}/P_{in}\;=\;1\;-\;P_{loss}/P_{in}.$$

In Equation (16), η is the transmission efficiency, $P_{out}$ is output power, $P_{in}$ is input power, and $P_{loss}$ is defined in Equation (15). At very small output power, subtracting two similar measured powers can produce large relative uncertainty, so direct output-torque measurement and a complete uncertainty budget are preferable. The mesh term in Equation (15) depends on load distribution, sliding velocity, surface topography, and lubricant state; microscale surface-sliding simulation can clarify the corresponding local mechanisms [36]. Support friction can dominate when teeth are lightly loaded. In liquid environments, $P_{fluid}$ rises with speed and exposed area; in freely suspended gears it may be the principal sink. Thus, reporting a single apparent friction coefficient without the architecture and medium rarely supports transferable design conclusions.

### 6.3. Wear and Surface Protection

Wear mechanisms include adhesive transfer, abrasion by debris, brittle fracture, fatigue, tribochemical reaction, and coating removal. A normalized wear measure may be stated as Equation (17):

(17) $$k_{w}\;=\;\Delta V/(W\;s).$$

In Equation (17), $k_{w}$ is the specific wear coefficient, ΔV is the lost material volume, W is the applied normal load, and s is the sliding distance. Equation (17) is useful only when ΔV, W, and s are meaningfully defined. Intermittent multi-tooth engagement and uncertain contact load complicate its application to a complete gear train. Direct observation of debris, tooth rounding, coating failure, and changes in torque or transmission error should therefore accompany a wear coefficient. Silicon studies reveal transitions between mild and severe damage [7], while conformal diamond-like carbon coatings can improve MEMS wear performance if coverage and adhesion are maintained [14].

Self-assembled monolayers reduce surface energy and can mitigate stiction, but molecularly thin protection has limited tolerance to defects and repeated contact. Damage mapping on functionalized microgears shows that local loss of the layer can initiate heterogeneous behavior [8]. Coating selection must consequently include deposition conformity, added thickness, residual stress, release compatibility, and endurance, not merely an initial friction value.

### 6.4. Lubrication and Environment

Continuous liquid films are difficult to sustain in lightly loaded, low-speed microcontacts. The film parameter in Equation (18) indicates the degree of asperity separation:

(18) $$\Lambda \;=\;h_{min}/\surd ({R_{q1}}^{2}\;+\;{R_{q2}}^{2}).$$

In Equation (18), Λ is the lubricant film parameter, $h_{min}$ is the minimum film thickness, and $R_{q1}$ and $R_{q2}$ are the root-mean-square roughness values of the two contacting surfaces. For many microgears, Equation (18) predicts boundary or mixed lubrication because $h_{min}$ is small and normalized roughness is high. Patterned confinement can retain liquids on silicon and create localized lubrication without flooding the entire MEMS device [34]. Vapor-phase or molecular lubrication may reduce drag, but performance remains sensitive to humidity and chemistry. Experiments in different vapor environments show that adhesive and corrosive wear can change substantially with atmosphere [35].

Table 7 summarizes the principal environmental effects. The operating medium must be declared in both design requirements and test reports; transferring a dry-air result to humid, vacuum, or liquid operation is generally unjustified.

## 7. Transmission Performance and Experimental Characterization

### 7.1. Torque Budget and Starting Behavior

A functional transmission must satisfy the torque balance in Equation (19):

(19) $$T_{in}\;>\;T_{load,ref}\;+\;T_{mesh}\;+\;T_{support}\;+\;T_{fluid}\;+\;J_{ref}\;\alpha .$$

In Equation (19), $T_{in}$ is the input torque, $T_{load,ref}$ is the load torque reflected to the input side, $T_{mesh}$ is the torque loss associated with gear meshing, $T_{support}$ is the support or bearing loss torque, $T_{fluid}$ is the fluid-drag torque, $J_{ref}$ is the reflected inertia, and α is the angular acceleration. Equation (19) should be evaluated at start-up and steady operation. Starting torque often exceeds running torque because static adhesion, meniscus forces, and initial alignment must be overcome. High-precision, long-range starting-torque measurement has been demonstrated using carefully calibrated microforce sensing and displacement tracking [9]. Resistive microtorque methods likewise require calibration of the compliant element and readout chain [15]. A beam–membrane microforce sensor illustrates the sensitivity and structural design needed at this scale [37].

When a tangential force is measured at a known radius, torque follows Equation (20):

(20) $$T\;=\;F_{t}\;r.$$

In Equation (20), T is torque, $F_{t}$ is tangential force, and r is the effective force-application radius. The radius must include probe geometry and deformation when relevant, rather than simply being taken as the nominal pitch radius.

### 7.2. Efficiency and Loss Separation

Input and output powers are $P_{in}=T_{in}\omega_{in}$ and $P_{out}{=T}_{out}\omega_{out}$. In these relations, $P_{in}$ and $P_{out}$ are input and output powers, $T_{in}$ and $T_{out}$ are input and output torques, and $\omega_{in}$ and $\omega_{out}$ are input and output angular velocities. For a reducer, the output torque predicted from measured efficiency is expressed by Equation (21):

(21) $$T_{out}\;=\;\eta \;|i|\;T_{in}.$$

In Equation (21), $T_{out}$ is output torque, η is transmission efficiency, |i| is the absolute value of the transmission ratio, and $T_{in}$ is input torque. Equation (21) is valid only when the sign convention is separated from torque magnitude and the system is in a defined operating state. Measuring $T_{in}$ without the gear train, then with successive components, can isolate support and mesh losses. Such subtraction must use identical speed, environment, alignment, and thermal history. Microgear-train studies emphasize the need to follow power through the entire train rather than infer performance from tooth geometry alone [4].

### 7.3. Backlash, Transmission Error, and Speed Fluctuation

Backlash is a geometric clearance modified by runout, deflection, and thermal or environmental changes. A first-order transverse estimate is given by Equation (22):

(22) $$j_{t}\;\approx \;j_{t,0}\;+\;2\;\Delta a_{w}\;tan\;\alpha_{t}.$$

In Equation (22), $j_{t}$ is the transverse backlash after center-distance deviation, $j_{t,0}$ is the nominal transverse backlash, $\Delta a_{w}$ is the center-distance deviation, and $\alpha_{t}$ is the transverse pressure angle; tooth-thickness deviations must be added consistently. In this context, the operating center-distance deviation ${\Delta a}_{w}$ should include not only the static manufacturing or assembly error, but also the load-dependent displacement of the supported shafts caused by radial bearing clearance and support compliance. Consequently, backlash and transmission error should preferably be measured under both torque directions and, where possible, under unloaded and loaded conditions. This comparison helps distinguish tooth-thickness and profile effects from reversible shaft migration, bearing play, and support deformation. Axial clearance and angular misalignment should also be reported when they alter face overlap or generate measurable out-of-plane motion. Transmission error (TE) compares actual output position with the position predicted by the nominal ratio, as defined in Equation (23):

(23) $$TE(t)\;=\;\theta_{out}(t)\;-\;\theta_{in}(t)/i.$$

In Equation (23), TE(t) is the transmission error as a function of time, $\theta_{out}(t)$ is the measured output angular position, $\theta_{in}(t)$ is the measured input angular position, and i is the nominal transmission ratio. Equation (23) captures pitch and profile errors, eccentricity, compliance, and intermittent contact. Optical tracking is often appropriate because it avoids mechanical loading, but image resolution, frame rate, marker placement, and out-of-plane motion contribute uncertainty. The instantaneous speed ratio in Equation (24) is especially sensitive to numerical differentiation and should be filtered without suppressing genuine tooth-passing fluctuations.

(24) $$i_{inst}(t)=\omega_{in}(t)/\omega_{out}(t).$$

In Equation (24), $i_{inst}(t)$ is the instantaneous transmission ratio, while $\omega_{in}$(t) and $\omega_{out}$(t) are the instantaneous input and output angular velocities.

### 7.4. Lifetime, Failure, and Validation Levels

Microscale lifetime cannot be represented by cycle count alone. Failure may be defined by fracture, seizure, loss of coating, unacceptable wear, increased starting torque, reduced efficiency, or excessive TE. A test should therefore record a state vector such as Equation (25), not only whether rotation continues:

(25) $$S_{N_{c}}\;=\;\{T_{start},\;\eta ,\;TE,\;j,\;\Delta V,\;debris\}.$$

In Equation (25), $S_{N_{c}}$ is the functional state after $N_{c}$ cycles, $T_{start}$ is starting torque, η is efficiency, TE is transmission error, j is backlash, ΔV is wear volume, and debris denotes the observed debris state. Table 8 proposes four levels of validation. The hierarchy distinguishes a fabricated gear from a demonstrated transmission and prevents visual rotation from being treated as evidence of loaded power transmission.

### 7.5. Measurement Influence and Minimum Test Matrix

The measuring system must be included in the mechanical model. Equation (26) defines a simple disturbance ratio:

(26) $$\Gamma_{T}\;=\;|T_{instr}|/|T_{trans}|.$$

In Equation (26), $\Gamma_{T}$ is the torque-disturbance ratio, $T_{instr}$ is the torque introduced by the instrument, and $T_{trans}$ is the characteristic transmitted torque. When $\Gamma_{T}$ is not negligible, the instrument changes the operating point. Probe stiffness, cable torque, bearing preload, optical trapping forces, and fluid motion caused by the measurement setup should be calibrated or bounded. Combined standard uncertainty may be estimated by Equation (27):

(27) $$u_{c}(y)\;=\;\surd [\Sigma \;{c_{i}}^{2}\;u^{2}(x_{i})].$$

In Equation (27), $u_{c}(y)$ is the combined standard uncertainty of the measurand y, $c_{i}$ is the sensitivity coefficient associated with input quantity $x_{i}$, and $u(x_{i})$ is the standard uncertainty of $x_{i}$. Table 9 presents a minimum functional test matrix. It is intentionally compact so that it can be applied to both MEMS gears and precision microgears while preserving comparability.

### 7.6. Preliminary Concept for a Reference Functional Test Artifact

**Figure 2:**
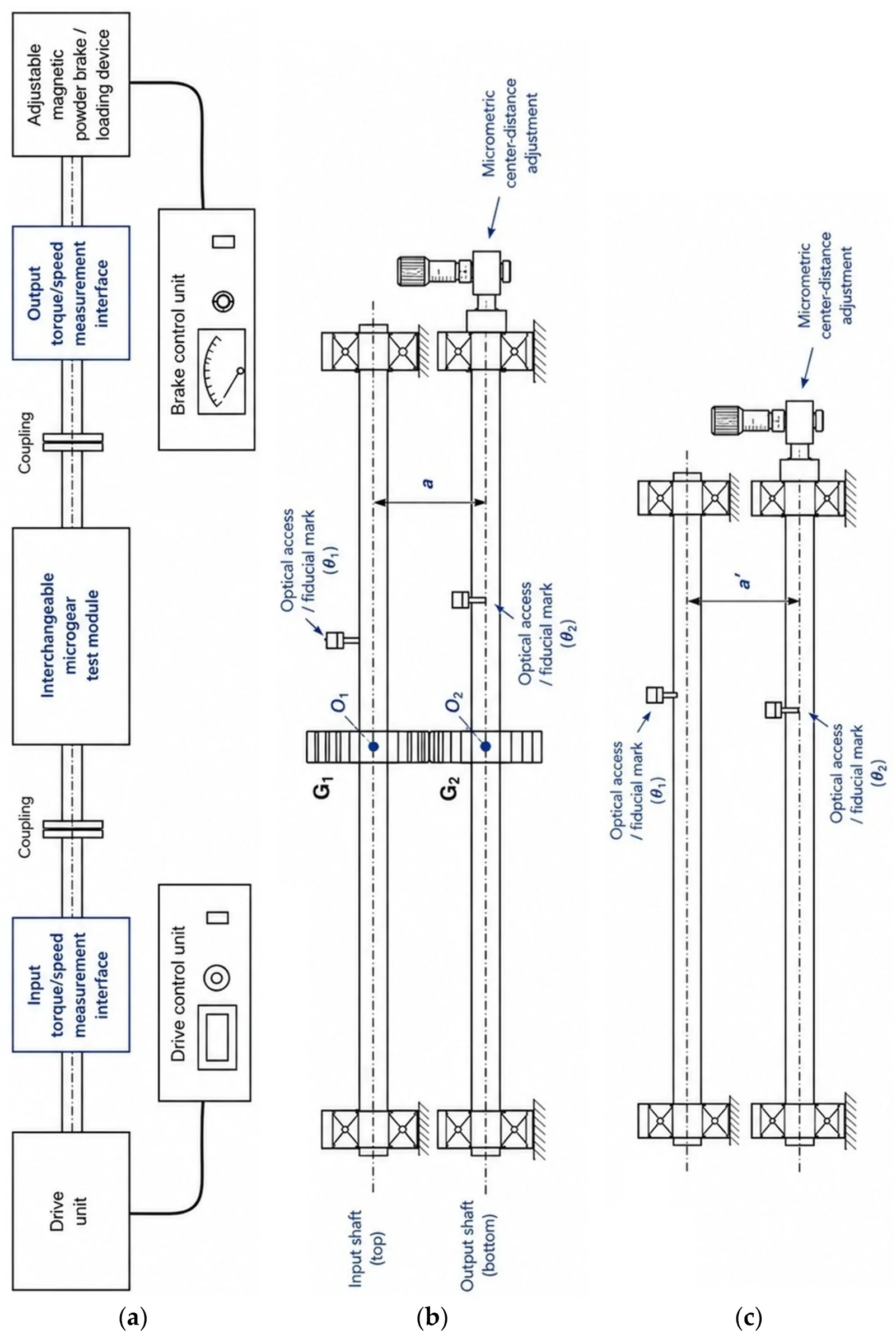
Preliminary concept of a reference functional test artifact for microgear transmissions: (**a**) overall arrangement of the test bench, including the controlled drive unit, input measurement interface, interchangeable microgear test module, output measurement interface, and adjustable loading device; (**b**) detail of the interchangeable module containing the single-stage external spur-gear pair mounted on two independently supported parallel shafts with adjustable center distance and optical access; (**c**) blank calibration configuration used to quantify support and instrumentation losses without tooth engagement.

The absence of common functional test artifacts limits the comparability of reported microgear results, particularly when different studies employ dissimilar geometries, supports, loading arrangements, environmental conditions, and measurement systems. As an initial step toward more reproducible and eventually standardized testing, a preliminary reference artifact is proposed in this section. The configuration is intended as a practical basis for interlaboratory comparison and protocol development rather than as a formal standard. The overall arrangement of the proposed test bench and the principal features of its interchangeable microgear test module are illustrated schematically in Figure 2.

The proposed artifact consists of a single-stage external spur-gear pair mounted on two parallel shafts with a controlled center distance. As shown in Figure 2a, the gear pair is incorporated into a functional test bench comprising a controlled drive unit, input and output measurement interfaces, coupling elements, and an adjustable loading device. This architecture is selected because it is planar, directly observable, compatible with several microscale manufacturing routes, and sufficiently simple to separate the effects of tooth geometry, surface condition, support friction, alignment, and measurement disturbance. More complex architectures, such as planetary, worm, or compound trains, introduce additional contacts and support elements that can obscure the origin of measured losses and transmission errors.

An indicative baseline geometry is defined by standard involute teeth with a transverse pressure angle of 20°, a module of 0.05 mm, and equal tooth numbers of 20 and 20. The resulting pitch diameters are 1.0 mm, the nominal center distance is 1.0 mm, and the ideal transmission ratio is 1:1. A nominal face width of 0.25 mm is proposed as a compromise between structural stiffness, process compatibility, and optical accessibility. The corresponding reference geometry and the arrangement of the two independently supported parallel shafts are detailed in Figure 2b. These values are intended to anchor comparison rather than restrict alternative geometries. Scaled variants may therefore be introduced, provided that all dimensions, tolerances, surface parameters, and operating conditions are explicitly reported.

The artifact should provide controlled and repeatable shaft positioning, direct optical access to both gears, and clearly defined input and output interfaces. The detailed interchangeable module shown in Figure 2b should permit independent support of the input and output shafts, micrometric adjustment of the center distance, and rapid replacement of the tested microgear pair. Fiducial marks placed on the gears or shafts should allow noncontact measurement of angular position, transmission error, backlash, and instantaneous speed ratio. The output shaft should include a calibrated loading interface, such as a microlever, micro-pulley, or equivalent torque-application feature. The input interface should permit either prescribed angular speed or prescribed torque, depending on the available actuation and sensing arrangement.

The input and output powers are evaluated as $P_{\mathrm{in}}=T_{\mathrm{in}}\omega_{\mathrm{in}}$ and $P_{\mathrm{out}}=T_{\mathrm{out}}\omega_{\mathrm{out}}$, where $P_{\mathrm{in}}$ and $P_{\mathrm{out}}$ are the input and output powers, $T_{\mathrm{in}}$ and $T_{\mathrm{out}}$ are the corresponding torques, and $\omega_{\mathrm{in}}$ and $\omega_{\mathrm{out}}$ are the corresponding angular velocities. Based on these quantities, the transmission efficiency is defined as the ratio of output power to input power and is expressed by Equation (28):

(28) $$\eta =\frac{P_{out}}{P_{in}}=\frac{T_{out}\omega_{out}}{T_{in}\omega_{in}}.$$

When torque is obtained from a measured reaction force acting through a calibrated lever arm, the corresponding relation is

(29) T=Fl,

where *F* is the measured reaction force and *l* is the effective lever-arm length. The dimensions, stiffness, calibration, and alignment of the reaction system should be included in the uncertainty budget because their influence may become significant relative to the small torque levels transmitted by the microgear pair.

A blank configuration should also be included, in which the two shafts and their supports operate under the same alignment, speed, and environmental conditions but without tooth engagement. This calibration arrangement is illustrated conceptually in Figure 2c. This configuration would allow support and instrumentation losses to be quantified independently from mesh losses. Such separation is particularly important at the microscale because bearing friction, compliant suspension forces, probe forces, and cable torques may represent a significant fraction of the transmitted torque. Depending on the construction of the test module, the blank condition may be obtained by removing the tested gears, disengaging the mesh through a controlled center-distance adjustment, or using equivalent inertial elements while preserving the shaft-support and measurement configuration.

The reference artifact should be tested using the functional matrix introduced in Table 9. At a minimum, the protocol should include dimensional and surface characterization, starting-torque measurements, steady-load testing, motion-quality assessment, endurance testing, and instrument verification. The primary reported quantities should include input and output torque, angular speed, efficiency, backlash, transmission error, starting-torque distribution, failed-start frequency, and the evolution of the functional state vector defined in Equation (25). The calibration and uncertainty budget should include the torque-disturbance ratio defined in Equation (26).

Dry air under controlled temperature and relative humidity may be adopted as the baseline environment. Tests conducted in humid air, vacuum, immersed liquids, or confined lubricants should be treated as declared extensions of the reference condition. Dwell time before start-up, approach direction, duty cycle, load spectrum, sampling frequency, and filtering procedures should also be specified because these variables can materially influence measured microscale behavior.

The proposed artifact is deliberately simple and modular. Its main purpose is to provide a common physical and methodological reference that can be manufactured using different processes and materials while preserving comparable geometry, interfaces, loading conditions, and reported outputs. Round-robin studies based on such an artifact could quantify interlaboratory variation, distinguish process-related deviations from measurement-related effects, and support the gradual development of more rigorous functional test protocols for microgear transmissions. Together, the reference geometry summarized in Table 10 and the modular test arrangement illustrated in Figure 2 define a preliminary physical basis for comparable functional testing without prescribing a unique manufacturing, actuation, loading, or instrumentation solution.

## 8. Applications of Microgear-Based Transmission Systems

### 8.1. MEMS Positioning, Timing, and Motion Conversion

Microgears can distribute motion among MEMS elements, reduce actuator speed, synchronize shutters or indicators, and convert oscillatory actuation into intermittent rotation. Silicon compatibility favors monolithic planar assemblies, but usable output is limited by support friction and surface degradation. In these applications, repeatable indexing and low starting torque may be more important than peak efficiency.

### 8.2. Micropumps and Fluidic Systems

Gear micropumps translate tooth-space displacement into flow. Their theoretical flow rate is represented by Equation (30):

(30) $$Q_{th}\;=\;V_{d}\;n.$$

In Equation (30), $Q_{th}$ is the theoretical flow rate, $V_{d}$ is the displacement per revolution, and n is the rotational speed. Actual flow is lower because of leakage, incomplete filling, compliance, and recirculation. Volumetric efficiency and hydraulic power are given by Equation (31):

(31) $$\eta_{v}\;=\;Q/Q_{th},\;\;\;P_{h}\;=\;\Delta p\;Q.$$

In Equation (31), $\eta_{v}$ is volumetric efficiency, Q is actual flow rate, $Q_{th}$ is theoretical flow rate, $P_{h}$ is hydraulic power, Δp is pressure rise, and Q is, again, the actual flow rate through the pump. Equation (31) shows that a pump must be evaluated by pressure–flow performance and mechanical input, not by visible rotation alone. A compact LIGA-based micropumping system demonstrates how gears, housing, and fluidic interfaces must be co-designed [40]. Liquid can lubricate the mesh, but it also adds viscous drag and imposes material-compatibility constraints.

### 8.3. Miniature Robots and Ultrasonic-Motor Drives

Miniature robots require high torque density, compact packaging, and tolerance to intermittent impacts. A millimeter-scale rolling microrobot driven by a microgeared ultrasonic motor demonstrates a complete chain from high-speed actuation through reduction to locomotion [39]. Such systems expose the reducer to shock, assembly misalignment, and duty-cycle heating that isolated gear tests may miss. Application-level validation should therefore include blocked-output events and repeated acceleration.

### 8.4. Reconfigurable and Externally Driven Micromachines

Magnetic, optical, and optoelectronic actuation eliminates conventional shafts and bearings, enabling assembly inside confined spaces or liquids. Femtosecond-written magnetic gears can be driven as a pair [3], while optoelectronic tweezers assemble and reconfigure multi-component micromachines [13]. Colloidal geared metamachines extend this principle to cooperative, topology-dependent motion [38]. The engineering metric shifts from shaft efficiency alone to controllability, synchronization, confinement stability, and the ratio of useful output to field input.

### 8.5. Application-Level Comparison

Table 11 maps application classes to their dominant requirements. It reinforces that a universally optimal microgear does not exist; the correct architecture depends on which failure or loss mechanism is least tolerable.

## 9. Design Guidelines, Research Gaps, and Future Directions

### 9.1. Scale-Aware Design Workflow

The literature supports a concurrent workflow. First, define the output motion, torque, speed, duty cycle, life, and environment. Second, choose an actuation and architecture whose ideal ratio can meet those needs with margin. Third, select a material–process pair and replace nominal geometry with expected as-manufactured geometry. Fourth, allocate a torque budget using Equation (19), including adhesion, supports, and fluid drag. Fifth, plan surface conditioning and packaging. Finally, design measurement and endurance tests before committing inaccessible or irreversible fabrication steps.

A useful torque-margin indicator is defined by Equation (32):

(32) $$M_{T}\;=\;T_{in,available}/T_{resist,max}.$$

In Equation (32), $M_{T}$ is the torque-margin indicator, $T_{in,available}$ is the available input torque, and $T_{resist,max}$ is the maximum resisting torque, including worst-case reflected load and parasitic torques. Values only slightly above unity are unlikely to tolerate process variation, aging, or environmental change. Equation (32) should be evaluated statistically when starting torque or fabrication deviations are distributed. Table 12 consolidates the recommended indicators and corresponding design responses.

**Figure 3:**
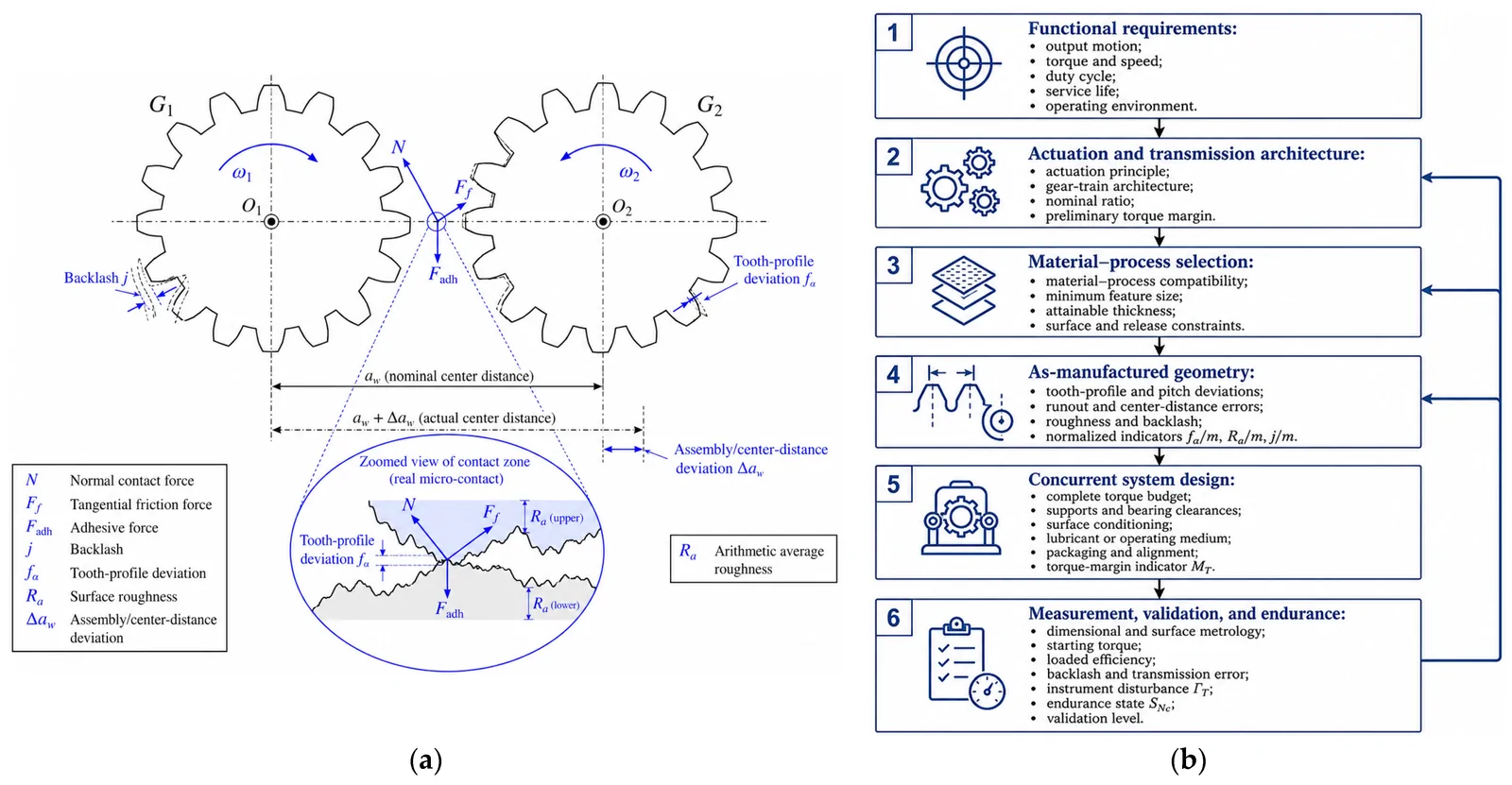
Microscale meshing and scale-aware design methodology: (**a**) schematic representation of external microgear engagement, showing the principal geometric, surface, and contact quantities that influence the real tooth interaction; (**b**) proposed concurrent scale-aware design workflow linking functional requirements, actuation and transmission architecture, material–process selection, as-manufactured geometry, system-level torque and surface design, measurement, validation, and endurance feedback.

The main physical factors governing microscale tooth engagement and the corresponding scale-aware design workflow are summarized schematically in Figure 3. Figure 3a emphasizes that nominal involute meshing must be considered together with center-distance deviation, backlash, profile error, roughness, friction, and adhesion. Figure 3b translates these coupled effects into a concurrent design sequence linking functional requirements, architecture, material and process selection, as-manufactured geometry, torque and surface design, measurement, and endurance validation.

### 9.2. Research Gaps

Four gaps recur. First, standardized functional test artifacts and protocols are missing. ISO flank tolerances [6] and microgear terminology [5] are valuable, but they do not prescribe a loaded microscale efficiency or endurance test. Second, process papers often stop after dimensional inspection, whereas tribology papers use simplified contacts that omit tooth kinematics. Third, long-duration data under controlled humidity, vacuum, or liquid conditions remain scarce. Fourth, uncertainty and instrument disturbance are not reported consistently, limiting comparisons of very small torques and losses.

Future studies should publish machine-readable geometry and operating data, including as-built profiles, surface statistics, alignment, environment, load, speed, sampling, calibration, and failure criteria. Round-robin measurements on common gear artifacts would reveal inter-laboratory variation. In situ optical, electrical, or acoustic monitoring could connect individual contact events to torque fluctuations and progressive damage.

### 9.3. Emerging Opportunities

Additive microfabrication, functional composites, and field-driven assembly expand the feasible design space beyond planar involute gears [3,11,13,38]. Their promise lies in integrated bearings, compliant features, magnetic domains, and reconfigurable topology. Progress will depend on treating these capabilities as transmission-system variables rather than fabrication demonstrations. Data-driven process compensation may reduce systematic profile errors, while digital twins based on measured geometry can connect fabrication, contact simulation, and lifetime prediction.

Data-driven process compensation can provide a direct connection between manufacturing conditions and the resulting functional geometry. Process parameters, such as lithographic exposure, electroforming conditions, tool or electrode trajectories, forming loads, molding and sintering conditions, laser-writing paths, and layer-by-layer fabrication settings, can be associated with measured deviations in tooth profile, pitch, runout, thickness, edge radius, and surface texture. Statistical, machine-learning, or hybrid physics-informed models may then identify systematic and repeatable error patterns that are not adequately represented by nominal process tolerances alone. The resulting error maps can be used to modify the mask geometry, tool path, electrode trajectory, mold cavity, or exposure strategy before subsequent fabrication [19,20]. The objective is not merely to reduce absolute dimensional deviations, but to reduce the normalized quantities introduced in Equation (7), including $\frac{f_{\alpha }}{m}$, $\frac{R_{a}}{m}$, and $\frac{j}{m}$, because these quantities more directly describe the influence of manufacturing errors, surface roughness, and backlash relative to tooth size.

Such compensation should be implemented as an iterative manufacturing–measurement loop. After an initial batch is produced, the as-manufactured profiles and surface characteristics are measured and compared with the intended geometry. The observed deviations are then related to the corresponding process inputs, and a corrected geometry or process parameter set is generated for the next batch. Repetition of this sequence can progressively reduce systematic errors while also revealing the residual stochastic variation that cannot be removed through deterministic compensation [19,20]. Reporting both the compensated mean geometry and the remaining batch-to-batch dispersion would therefore be essential, particularly when the available torque margin is small and individual deviations may alter contact continuity, starting behavior, or load sharing.

A digital twin for a microgear transmission should extend beyond a simulation based on nominal CAD geometry. It should represent a specific manufactured specimen or statistically defined production batch by incorporating measured tooth profiles, thickness variation, surface parameters, material properties, shaft and bearing characteristics, center-distance deviation, alignment, operating medium, load, and speed. Using this specimen-specific information, the model could estimate tooth-contact evolution, load distribution, intermittent engagement, support and mesh losses, starting torque, transmission error, efficiency, and the locations most susceptible to wear or coating damage. The measured geometry would therefore become an active model input rather than a final inspection result that remains disconnected from performance prediction. Digital-twin-assisted updating of gear-system models provides a methodological basis for this type of physical–virtual integration [21].

The digital twin should also be updated using functional measurements obtained from the test procedures proposed in Section 7. Starting torque, input and output torque, angular speed, backlash, transmission error, temperature, and the functional state variables monitored during endurance testing can be used to calibrate uncertain contact, friction, support, and degradation parameters. Physical–virtual signal interaction and parameter-sensitive updating have already been applied to improve the fidelity of gearbox dynamic models [21]. As additional operating data become available, the model can be updated to distinguish geometric errors from surface degradation, environmental effects, and instrumentation disturbance. In this way, the twin may evolve from an initial as-manufactured model into an as-operated and, subsequently, an as-degraded representation of the transmission [22].

The combined use of data-driven compensation and digital twins creates a closed information loop linking process parameters, measured geometry, predicted contact behavior, loaded testing, and subsequent process correction. This loop can address several of the gaps identified in Section 9.2. It can connect process-oriented studies to system-level performance, replace idealized geometry with measured geometry, support reproducible compensation of systematic manufacturing errors, and relate endurance data to progressive changes in torque, efficiency, transmission error, and surface condition [19,20,21,22]. It can also propagate measurement uncertainty through the model so that predicted performance is reported with confidence bounds rather than as a single deterministic value. Such uncertainty-aware predictions are particularly important when instrument torque, support losses, or environmental effects are comparable to the useful transmitted torque.

At the production level, the same framework could support acceptance criteria based on predicted functional performance rather than dimensional compliance alone. A fabricated gear that satisfies an absolute profile tolerance may still exhibit excessive normalized roughness, insufficient torque margin, or unacceptable transmission error under the intended environment. Conversely, a measured deviation that appears large in isolation may have limited functional influence under a specific load and speed range. Linking inspection data to a calibrated digital twin would therefore allow dimensional and surface measurements to be interpreted according to their predicted consequences for the complete transmission [21,22].

The most consequential opportunity is methodological: researchers can report the complete energy and uncertainty chain from actuator to output. Once starting torque, steady losses, motion quality, and degradation are measured under a declared environment, different materials and architectures can be compared on functional grounds. This would move the field from successful rotation toward predictable mechanical power transmission.

## 10. Conclusions

Microgear transmissions preserve the geometric logic of conventional gearing but not its balance of forces, errors, losses, and measurement effects. Torque capacity decreases approximately with the cube of scale, inertia decreases with the fifth power, and surface-to-volume ratio increases. Consequently, adhesion, support friction, viscous drag, coating integrity, and normalized manufacturing errors can dominate behavior.

No manufacturing route is universally superior. Silicon micromachining favors integration; electroforming provides thicker metallic structures; molding and forming support replication; micro-EDM and cutting provide functional metallic prototypes; ultrashort-pulse laser ablation enables non-contact processing of difficult-to-machine metallic and ceramic microcomponents; and direct writing enables complex or reconfigurable devices. Each route creates a characteristic combination of geometry, surface condition, and assembly constraints that must enter the transmission design.

The literature is rich in fabricated gears but thinner in loaded, uncertainty-qualified, long-duration transmission data. The validation hierarchy in Table 8 and the test matrix in Table 9 provide a practical basis for strengthening future claims. A robust microgear system should be designed concurrently across geometry, process, material, surface, environment, actuation, load path, and measurement.

The present review and the proposed framework also have several limitations. The study is a critical engineering synthesis rather than a systematic statistical meta-analysis, because the available datasets differ substantially in characteristic size, material, manufacturing route, actuation, load, speed, operating medium, and reported uncertainty. The descriptive evidence distribution therefore characterizes the corpus assembled for this review and should not be interpreted as an exhaustive bibliometric representation of the entire microgear field. Assigning each study to one dominant technical category also simplifies contributions that may span several adjacent domains. Furthermore, the scaling relations and normalized indicators are first-order engineering tools based on declared assumptions and cannot replace geometry-, material-, process-, and application-specific analyses. The proposed validation hierarchy, functional test matrix, scale-aware workflow, and reference test artifact have not yet been verified through coordinated interlaboratory experiments and should therefore be regarded as preliminary methodological recommendations rather than formal standards.

Future work should consequently prioritize round-robin manufacture and testing of common reference artifacts across different materials, fabrication routes, laboratories, and measurement systems. Such studies should establish the repeatability, reproducibility, and uncertainty limits of starting-torque, loaded-efficiency, backlash, transmission-error, and endurance measurements under controlled dry-air, humid, vacuum, and liquid environments. Particular attention should be given to shaft- and planet-pin positioning, bearing clearances, support compliance, and instrument-induced disturbance because these effects may be comparable to the useful transmitted torque. Machine-readable publication of as-manufactured geometry, surface statistics, alignment, operating conditions, calibration data, and failure criteria would support cross-study comparison. Data-driven process compensation and digital twins should subsequently be validated using measured geometry and functional endurance data so that process corrections and performance predictions can be assessed against experimentally observed transmission behavior and degradation.

Under that systems view, microgears can progress from visually compelling demonstrators to predictable components for microsystems, fluidic devices, and miniature robots.

## Figures and Tables

**Table 1:** Eligibility criteria used for literature selection.

Criterion	Included	Excluded
Physical scope	Fabricated micro/miniature gears, gear trains, or directly relevant tooth-contact specimens	Macroscale gearing without a microscale argument
Technical scope	Design, process, material, metrology, tribology, performance, or application evidence	Unrelated microactuators or mechanisms without gear contact
Evidence	Quantitative experiments, validated models, process characterization, or standards	Unverifiable promotional descriptions
Language and access	English-language records with sufficient methodological detail	Records lacking enough information for technical interpretation
Temporal scope	Foundational studies and relevant work available through June 2026	Superseded duplicates when a complete version was available

**Table 2:** Descriptive classification of the primary microgear-specific technical evidence included in the review.

Analysis Dimension	Category or Validation Level	References	Number	Share
**Dominant technical emphasis**	Manufacturing, process, and geometry	[10,11,24,25,26,27,28,29,30,31,32]	11	39.3%
	Tribology and surface/contact behavior	[7,8,14,33,34,35,36]	7	25.0%
	Metrology and instrumentation	[9,12,15,37]	4	14.3%
	Functional transmission and system-level application	[3,4,13,38,39,40]	6	21.4%
	**Total primary technical studies**	**—**	**28**	**100%**
**Highest validation level**	Level 0—fabricated geometry and dimensional inspection	[10,11,12,24,25,26,27,28,29,30,31,32]	12	66.7%
	Level 1—unloaded rotation or demonstrated meshing	[3,38]	2	11.1%
	Level 2—quantified functional or loaded operation	[4,13,39,40]	4	22.2%
	Level 3—complete quantified transmission performance	—	0	0%
	Level 4—endurance and failure analysis	—	0	0%
	**Total eligible studies**	**—**	**18**	**100%**

**Table 3:** Working dimensional classification adopted in this review.

Class	Indicative Dimensions	Typical Technologies and Remarks
MEMS-scale microgear	Module commonly below 0.02 mm; overall size from tens of micrometers to about 1 mm	Surface/bulk micromachining, lithography–electroforming, two-photon fabrication; surface forces often dominant
Precision microgear	Module approximately 0.02–0.10 mm; diameter commonly below several millimeters	Micro-cutting, micro-EDM, electroforming, molding, forming; functional power transmission feasible
Miniature gear	Module approximately 0.10–0.30 mm; diameter commonly millimetric to centimetric	Precision machining, molding, forming; conventional gear concepts remain useful, but relative errors are large

**Table 4:** Scaling relationships for geometrically similar gears.

Quantity	Approximate Dependence on λ	Design Implication
Length, module, radius	λ	Nominal geometry scales directly
Contact/root area	$\lambda^{2}$	Force capacity decreases rapidly
Mass and torque capacity	$\lambda^{3}$	Small parasitic torques become important
Mass moment of inertia	$\lambda^{5}$	Fast response but high disturbance sensitivity
Surface-to-volume ratio	$\lambda^{-1}$	Surface chemistry and environment gain influence

**Table 5:** Comparative assessment of microgear transmission architectures.

Architecture	Principal Advantage	Dominant Microscale Difficulty	Best-Suited Use
External spur	Planar, inspectable, process compatible	Center-distance and shaft-position control, bearing clearance, backlash, and edge defects	General planar transmission
Internal	Compact, same rotation direction	Interference and tool/process access	Coaxial compact stages
Planetary	Coaxial high ratio and load sharing	Planet phasing, pin-position errors, bearing clearances, and carrier friction	Compact reducers
Compound planar	Large ratio using repeated geometry	Accumulated shaft-position errors, eccentricity, bearing clearances, backlash, and losses	Low-load multistage reduction
Helical/bevel	Smooth contact or axis redirection	3D fabrication, alignment, axial load	Packaging-driven layouts
Worm	High single-stage ratio	Sliding loss and wear	Intermittent low-power positioning
Compliant/strain-wave	Very high ratio, coaxial	Fatigue and thin-wall manufacture	Specialized precision reduction
Free/reconfigurable gears	Assembly and topology can change	Confinement, control, weak output coupling	Microfluidic and optical systems

**Table 6:** Comparison of materials and manufacturing technologies for microgear.

Route	Typical Materials	Strengths	Principal Limitations
Silicon micromachining	Single/polycrystalline silicon	Batch precision; MEMS integration	Brittleness; thin planar geometry; poor unprotected tribology
Lithography + electroforming	Ni and Ni alloys	Fine planar profiles; useful thickness	Taper, residual stress, thickness nonuniformity
Micro-PIM	Metals; zirconia; composites	Replication and batch production	Shrinkage, warpage, debinding/sintering defects
Microforming/blanking	Ductile metals	High rate; good material use	Burrs, die wear, grain and friction size effects
Micro-cutting/EDM	Conductive metals and hard alloys	Functional prototypes; material flexibility	Tool/electrode limits, recast or burrs, lower throughput
Two-photon/direct writing	Photopolymers; functional composites	Complex 3D shapes; integrated assembly	Creep, anisotropy, roughness, limited load
Ultrashort-pulse laser ablation	Metals, alloys, PZT, and other technical ceramics	Non-contact processing; no tool wear; high-resolution contouring; three-dimensional access; rapid prototyping	Parameter-sensitive taper and edge quality; redeposition and debris; roughness or microcracking; limited batch throughput

**Table 7:** Influence of operating environment on microgear tribology.

Environment	Potential Benefit	Principal Risk and Design Response
Dry air	Simple packaging and observation	Oxidation, debris, variable adsorbates; control cleanliness and humidity
Humid air	Possible surface passivation	Capillary stiction and tribochemistry; use low-energy surfaces and humidity tests
Vacuum	No capillary condensation; low fluid drag	Desorption, cold welding, limited heat removal; qualify coatings in vacuum
Immersed liquid	Debris removal, damping, possible lubrication	Viscous drag and swelling/corrosion; include fluid torque in the budget
Confined lubricant	Localized film with limited global drag	Retention, contamination, aging; design reservoirs and confinement

**Table 8:** Levels of experimental validation for microgear systems.

Level	Evidence	Minimum Claim Supported
0	Fabricated geometry and dimensional inspection	Manufacturability of a gear feature
1	Unloaded rotation or meshing over a limited interval	Kinematic feasibility
2	Measured torque/speed under a defined load and environment	Functional power transmission
3	Efficiency, TE/backlash, uncertainty, and repeated tests	Quantified transmission performance
4	Endurance with periodic state measurements and failure analysis	Reliability for an operating envelope

**Table 9:** Recommended test matrix for functional microgear characterization.

Test Group	Controlled Variables	Reported Outputs
Geometry and assembly	Temperature, datum, instrument, filtering, assembly procedure, support preload	Module, pitch/profile deviation, shaft-center coordinates, pin-position errors, operating center-distance deviation ${\Delta a}_{w}$, radial and axial bearing clearances, shaft and gear runout, angular misalignment, face overlap, thickness, roughness, support stiffness or preload, uncertainty
Start-up	Dwell time, humidity/medium, approach direction, torque direction	Starting-torque distribution, failed starts, hysteresis, shaft-center migration at start-up
Steady load	Input speed, output load, torque direction, environment, support preload	$T_{in}$, $T_{out}$, ratio, efficiency, temperature, load-dependent shaft-center displacement
Motion quality	Load and speed range, torque direction, unloaded/loaded condition, sampling rate	Backlash, TE, speed fluctuation, reversible shaft migration, out-of-plane motion, repeatability
Endurance	Duty cycle, load spectrum, environment, periodic clearance and alignment checks	State vector $S_{N_{c}}$ versus cycles, bearing-clearance and alignment evolution, debris, failure mode
Instrument check	Calibration and blank configuration	$\mathrm{Resolution},\;\mathrm{drift},\;\Gamma_{T}$, combined uncertainty

**Table 10:** Indicative baseline specification for the proposed reference functional test artifact.

Parameter	Proposed Baseline
Transmission architecture	Single-stage external spur-gear pair
Tooth profile	Standard involute
Pressure angle	20°
Module	0.05 mm
Tooth numbers	20/20
Nominal transmission ratio	1:1
Pitch diameters	1.0 mm/1.0 mm
Nominal center distance	1.0 mm
Nominal face width	0.25 mm
Shaft arrangement	Two parallel shafts with controlled positioning
Motion measurement	Noncontact optical tracking using fiducial marks
Input measurement	Input torque and angular speed
Output measurement	Output torque and angular speed
Output loading	Calibrated microlever, micro-pulley, magnetic brake, or equivalent interface
Center-distance adjustment	Micrometric adjustment of one shaft-support assembly
Calibration configuration	Blank configuration without tooth engagement
Baseline environment	Dry air at controlled temperature and relative humidity
Minimum reported outputs	Starting torque, input/output torque, angular speed, efficiency, backlash, transmission error, repeatability, and uncertainty
Transmission architecture	Single-stage external spur-gear pair

**Table 11:** Application-level comparison of microgear-based systems.

Application	Primary Function	Dominant Requirement	Critical Validation
MEMS positioning	Indexing, timing, motion conversion	Low starting torque and repeatability	Bidirectional cycles, TE, stiction
Micropump	Fluid displacement	Sealing and pressure capability	Pressure–flow–power map and leakage
Microrobot drive	Torque multiplication for locomotion	Shock tolerance and compactness	Loaded motion, stall, duty cycle
Optical/magnetic assembly	Remote coordination	Controllability in a medium	Field-response map and synchronization
Metamachine	Collective reconfigurable behavior	Topology and interaction stability	Repeatable state transitions and useful output

**Table 12:** Recommended design indicators and responses.

Indicator	Meaning	Design Response
$f_{\alpha }/m$, $R_{a}/m$, j/m	Geometry and surface relative to tooth scale	Choose process and tolerances using normalized, not only absolute, values
$M_{T}$	Available-to-resistant torque margin	Increase actuator margin, reduce contacts/support loss, or reduce required load
$\Gamma_{T}$	Instrument disturbance relative to transmitted torque	Use noncontact methods or calibrate and model probe influence
Λ	Lubricant film relative to combined roughness	Select boundary coatings, confinement, viscosity, and speed deliberately
$S_{N_{c}}$	Evolution of functional state with cycles	Define failure thresholds and inspect periodically during endurance
Validation level	Strength of the experimental claim	Match publication/application claims to evidence in Table 8

## Data Availability

Data are contained within the article.

## Abbreviations

**Symbol/Notation**	**Definition**	**Unit**
a	Nominal center distance between the two shaft axes in the engaged microgear test configuration.	m
a′	Center distance between the two shaft axes in the blank calibration configuration.	m
b	Gear face width.	m
$c_{i}$	Sensitivity coefficient associated with the input quantity $x_{i}$ in the uncertainty model.	unit of y $/\mathrm{unit}\;\mathrm{of}\;x_{i}$
d	Pitch diameter.	m
debris	Observed debris state included in the functional-state vector.	—
F	Measured reaction force acting through the calibrated lever arm in the reference test artifact.	N
$F_{\mathrm{adh}}$	Adhesive contribution to the normal load.	N
$F_{f}$	Friction force.	N
$F_{t}$	Tangential force.	N
$F_{t,\mathrm{allow}}$	Allowable tangential tooth force.	N
$f_{\alpha }$	Absolute tooth-profile deviation.	m
$G_{1},\;G_{2}$	Test gears 1 and 2 in the interchangeable microgear module.	—
$h_{\min}$	Minimum lubricant-film thickness.	m
i	Transmission or speed ratio, as defined for the considered architecture.	—
$i_{12}$	Angular-speed ratio between gears 1 and 2.	—
$i_{12,\mathrm{ext}}$	External-mesh transmission ratio.	—
$i_{12,\mathrm{int}}$	Internal-mesh transmission ratio.	—
$i_{\mathrm{inst}}\left(t\right)$	Instantaneous transmission ratio as a function of time.	—
J	Mass moment of inertia.	$\mathrm{kg}\cdot m^{2}$
$J_{\mathrm{ref}}$	Inertia reflected to the input side.	$\mathrm{kg}\cdot m^{2}$
j	Linear backlash used in the normalized backlash indicator.	m
$j_{t}$	Transverse backlash after center-distance deviation.	m
$j_{t,0}$	Nominal transverse backlash.	m
$k_{w}$	Specific wear coefficient.	$m^{3}/(N\cdot m)$
L	Generic scaled or characteristic length.	m
$L_{0}$	Reference length corresponding to L.	m
l	Effective lever-arm length used for reaction-force torque measurement.	m
m	Gear module.	m
$M_{T}$	Available-to-resisting torque-margin indicator.	—
n	Rotational speed used in the micropump flow-rate relation.	$s^{-1}$ or $\mathrm{rev}\cdot s^{-1}$
N	Externally applied normal load in the friction relation.	N
$N_{c}$	Number of operating cycles; recommended replacement for N in the state-vector notation.	cycles
$O_{1},\;O_{2}$	Centers of gears $G_{1}$ and $G_{2}$ in the test-module schematic.	—
$P_{\mathrm{aux}}$	Auxiliary power loss associated with seals, confinement, actuation coupling, or measurement interfaces.	W
$P_{\mathrm{fluid}}$	Power loss due to fluid drag.	W
$P_{h}$	Hydraulic power.	W
$P_{\mathrm{in}}$	Input mechanical power.	W
$P_{\mathrm{loss}}$	Total mechanical power loss.	W
$P_{\mathrm{mesh}}$	Power loss associated with gear meshing.	W
$P_{\mathrm{out}}$	Output mechanical power.	W
$P_{\mathrm{support}}$	Power loss in shafts, bearings, or compliant supports.	W
Q	Actual volumetric flow rate through the micropump.	$m^{3}\cdot s^{-1}$
$Q_{\mathrm{th}}$	Theoretical volumetric flow rate of the micropump.	$m^{3}\cdot s^{-1}$
$R_{a}$	Arithmetic mean surface roughness.	m
$R_{q1},\;R_{q2}$	Root-mean-square roughness values of the two contacting surfaces.	m
r	Effective force-application radius.	m
$R_{e}$	Reynolds number.	—
s	Sliding distance used in the wear relation.	m
$S_{N_{c}}$	Functional state vector after $N_{c}$ operating cycles.	—
T	Torque; in Section 7.6, torque inferred from reaction force and lever arm.	N·m
$T_{\mathrm{allow}}$	Allowable torque capacity.	N·m
$T_{\mathrm{fluid}}$	Fluid-drag torque.	N·m
$T_{\mathrm{in}}$	Input torque.	N·m
$T_{\mathrm{in},\mathrm{available}}$	Available input torque.	N·m
$T_{\mathrm{instr}}$	Disturbance torque introduced by the measuring instrument; recommended replacement for $T_{\mathrm{inst}}$ in Equation (26).	N·m
$T_{\mathrm{load},\mathrm{ref}}$	Load torque reflected to the input side.	N·m
$T_{\mathrm{mesh}}$	Torque loss associated with gear meshing.	N·m
$T_{\mathrm{out}}$	Output torque.	N·m
$T_{\mathrm{resist},\max}$	Maximum resisting torque, including worst-case reflected load and parasitic torques.	N·m
$T_{\mathrm{start}}$	Starting torque.	N·m
$T_{\mathrm{support}}$	Torque loss associated with bearings or supports.	N·m
$T_{\mathrm{trans}}$	Characteristic transmitted torque.	N·m
TE(t)	Transmission error as a function of time.	rad
t	Time.	s
U	Characteristic fluid velocity.	$m\cdot s^{-1}$
$u\left(x_{i}\right)$	Standard uncertainty associated with input quantity $x_{i}$.	$\mathrm{unit}\;\mathrm{of}\;x_{i}$
$u_{c}\left(y\right)$	Combined standard uncertainty of the measurand y.	unit of y
$V_{d}$	Displacement volume per revolution of the micropump.	$m^{3}\cdot {\mathrm{rev}}^{-1}$
W	Applied normal load used in the wear relation.	N
$x_{i}$	Input quantity $x_{i}$ in the uncertainty model.	depends on quantity
Y	Tooth-form factor in the simplified tooth-root capacity relation.	—
y	Measurand y in the uncertainty model.	depends on measurand
z	Number of gear teeth.	—
$z_{1},\;z_{2}$	Tooth numbers of gears 1 and 2.	—
$z_{c}$	Circular-spline tooth number.	—
$z_{f}$	Flexspline tooth number.	—
$z_{\mathrm{gear}}$	Tooth number of the gear in a worm-gear pair.	—
$z_{r}$	Ring-gear tooth number.	—
$z_{s}$	Sun-gear tooth number.	—
$z_{w}$	Number of worm starts.	—
α	Angular acceleration.	$\mathrm{rad}\cdot s^{-2}$
$\alpha_{t}$	Transverse pressure angle.	rad or °
β	Normalized backlash indicator, j/m.	—
$\Gamma_{T}$	Torque-disturbance ratio.	—
${\Delta a}_{w}$	Center-distance deviation.	m
Δp	Pressure rise across the micropump.	Pa
ΔV	Lost material volume or wear volume.	$m^{3}$
$\varepsilon_{\alpha }$	Normalized tooth-profile-deviation indicator, $f_{\alpha }/m$.	—
η	Mechanical transmission efficiency.	—
$\eta_{v}$	Volumetric efficiency of the micropump.	—
$\theta_{\mathrm{in}}(t)$	Measured input angular position as a function of time.	rad
$\theta_{\mathrm{out}}\left(t\right)$	Measured output angular position as a function of time.	rad
$\theta_{1},\;\theta_{2}$	Angular positions obtained from the two optical fiducial marks in the test-module schematic.	rad
Λ	Lubricant-film parameter.	—
λ	Geometric scale factor.	—
μ	Coefficient of friction.	—
$\mu_{f}$	Dynamic viscosity of the operating fluid; recommended replacement for μ in Equation (8).	Pa·s
$\rho_{a}$	$\mathrm{Normalized}\;\mathrm{roughness}\;\mathrm{indicator},\;R_{a}/m$.	—
$\rho_{f}$	Fluid density.	$\mathrm{kg}\cdot m^{-3}$
$\sigma_{\mathrm{allow}}$	Allowable material stress.	Pa
$\omega_{1},\;\omega_{2}$	Angular velocities of gears 1 and 2.	$\mathrm{rad}\cdot s^{-1}$
$\omega_{s},\;\omega_{r},\;\omega_{c}$	Angular velocities of the sun gear, ring gear, and carrier, respectively.	$\mathrm{rad}\cdot s^{-1}$
$\omega_{\mathrm{in}},\omega_{\mathrm{out}}$	Input and output angular velocities.	$\mathrm{rad}\cdot s^{-1}$
Σ	Summation over the input quantities included in the uncertainty model.	—
0	Reference or nominal value.	—
1, 2	Gear, shaft, or measured-channel indices 1 and 2.	—
adh	Adhesive contribution.	—
allow	Allowable value.	—
aux	Auxiliary loss.	—
available	Available input quantity.	—
c	$\mathrm{Carrier}\;\mathrm{in}\;\omega_{c}$$;\;\mathrm{circular}\;\mathrm{spline}\;\mathrm{in}\;z_{c}$$;\;\mathrm{combined}\;\mathrm{value}\;\mathrm{in}\;u_{c}$.	—
d	Displacement per revolution.	—
ext	External gear mesh.	—
f	$\mathrm{Fluid}\;\mathrm{in}\;\rho_{f}$$\;\mathrm{and}\;\mu_{f}$$;\;\mathrm{friction}\;\mathrm{in}\;F_{f}$$;\;\mathrm{flexspline}\;\mathrm{in}\;z_{f}$.	—
fluid	Fluid-drag loss or torque.	—
gear	Gear member of a worm-gear pair.	—
h	Hydraulic quantity.	—
in	Input quantity.	—
inst	$\mathrm{Instantaneous}\;\mathrm{value},\;\mathrm{retained}\;\mathrm{for}\;i_{\mathrm{inst}}$ only.	—
instr	$\mathrm{Instrument}-\mathrm{induced}\;\mathrm{disturbance},\;\mathrm{recommended}\;\mathrm{for}\;T_{\mathrm{instr}}$.	—
int	Internal gear mesh.	—
load	Applied or reflected load.	—
max	Maximum value.	—
mesh	Gear-mesh contribution.	—
min	Minimum value.	—
out	Output quantity.	—
q1, q2	Root-mean-square roughness values of contacting surfaces 1 and 2.	—
r	$\mathrm{Ring}\;\mathrm{gear}\;\mathrm{in}\;\omega_{r}$$\;\mathrm{and}\;z_{r}$.	—
ref	Quantity reflected to the input side or reference value.	—
resist	Resisting torque.	—
s	$\mathrm{Sun}\;\mathrm{gear}\;\mathrm{in}\;\omega_{s}$$\;\mathrm{and}\;z_{s}$.	—
start	Start-up value.	—
support	Shaft, bearing, or compliant-support contribution.	—
t	Tangential or transverse quantity, depending on the symbol.	—
th	Theoretical quantity.	—
trans	Transmitted quantity.	—
v	Volumetric quantity.	—
w	$\mathrm{Worm}\;\mathrm{in}\;z_{w}$$\;\mathrm{or}\;\mathrm{wear}\;\mathrm{in}\;k_{w}$.	—
α	Tooth-profile quantity.	—
3D	Three-dimensional.	—
AGMA	American Gear Manufacturers Association.	—
ANSI	American National Standards Institute.	—
CAD	Computer-aided design.	—
DLC	Diamond-like carbon.	—
ISO	International Organization for Standardization.	—
LIGA	Lithographie, Galvanoformung, Abformung (lithography, electroforming, and molding).	—
MEMSs	Microelectromechanical systems.	—
micro-EDM	Micro-electrical discharge machining.	—
micro-PIM	Micro powder injection molding.	—
NEMSs	Nanoelectromechanical systems.	—
SI	International System of Units.	—
TE	Transmission error.	—
VDI	Verein Deutscher Ingenieure (Association of German Engineers).	—
